# Interactions between *Aeromonas caviae* and *Yersinia enterocolitica* isolated from a case of diarrhea: evaluation of antimicrobial susceptibility and immune response of infected macrophages

**DOI:** 10.3389/fmicb.2024.1328766

**Published:** 2024-04-24

**Authors:** Ana Fernández-Bravo, Gemma Recio, Maria José Figueras

**Affiliations:** ^1^Department of Basic Medical Sciences, Mycology and Environmental Microbiology Unit, Rovira i Virgili University, Reus, Spain; ^2^Pere Virgili Health Research Institute (IISPV), Reus, Spain; ^3^Laboratori Clínic ICS Camp de Tarragona-Terres de l'Ebre, Instituto Catalán de la Salud, Tarragona, Spain

**Keywords:** *Aeromonas caviae*, *Yersinia enterocolitica*, mixed infections, macrophages, immune response, antimicrobial susceptibility

## Abstract

*Aeromonas* species cause a wide spectrum of human diseases, primarily gastroenteritis, septicemia, and wound infections. Several studies have shown that about 40% of these cases involve mixed or polymicrobial infections between *Aeromonas* spp. and bacteria from other genera. However, the immune response of macrophages in front of the bacteria present in the mixed infections, as well as their impact on antimicrobial therapy, have not been investigated. This study evaluated the cell damage and immune response of the mouse macrophage BALB/c cell line (J774A.1) after performing a single and a mixed infection with a strain of *Aeromonas caviae* and *Yersinia enterocolitica*, both recovered from the same fecal sample from a patient with diarrhea. Macrophage cell damage was measured by the release of lactate dehydrogenase (LDH) while the immune response was evaluated studying the expression by RT-qPCR of six relevant immune-related genes. Additionally, the antimicrobial susceptibility pattern of the single and mixed strains in front of seventeen antibiotics was evaluated to determine the potential impact on the infection treatment. Macrophages infected with the mixture of the two strains showed a higher cell damage in comparison with the single infections and the immune-related genes, i.e., cytokines and chemokines genes (*TNF-α, CCL20*), and apoptotic and pyroptotic genes (*TP53 and IL-1β*) were overexpressed. After infection with the mixed cultures, an increase in the antimicrobial resistance was observed for ciprofloxacin, trimethoprim, chloramphenicol, gentamicin and ertapenem. This study increased the knowledge about the synergetic effect of the bacteria involved in mixed infection and on their potential impact on the treatment and evolution of the infection.

## Introduction

Some species of the genus *Aeromonas* are considered human opportunistic emerging pathogens because they cause a wide spectrum of diseases, mainly gastroenteritis, wound infections, and bacteremia (Janda and Abbott, 2010; Figueras and Beaz-Hidalgo, 2015; Fernández-Bravo and Figueras, 2020). Infections caused by *Aeromonas* may involve more than one type of bacteria within the same clinical sample either of the same genera of other genera (Lamy et al., 2009; Mosser et al., 2015; Fernández-Bravo and Figueras, 2020). For instance, a co-infection involving two strains of *Aeromonas hydrophila* which were isolated before and after amputation and surgical debridement from the wound site of a severe case of necrotizing fasciitis (NF) occurred in a previously healthy young girl (Grim et al., 2014; Ponnusamy et al., 2016). The interaction of the two involved *A. hydrophila* strains in relation to the outcome of the infection was investigated using different murine animal models and results demonstrated that the enhanced virulence of the mixed infection was related to the existence of specific virulence genes in both bacteria (Grim et al., 2014; Ponnusamy et al., 2016; Fernández-Bravo et al., 2019). Experimental mixed infections with different *Aeromonas* spp. and/or strains using other animal models like *Caenorhabditis elegans* (Mosser et al., 2015) and *Drosophila melanogaster* (Barraud et al., 2020; Robert et al., 2023) showed, in general, an increase of the virulence as determined by the survival experiment after infection with the two *Aeromonas* strains. However, Robert et al. (2023) showed that the enhanced virulence was strain dependent.

According to Lamy et al. (2009) polymicrobial infections with *Aeromonas* represents 42.3% of the clinical cases, while in a study included in the Ph.D. of Latif (2015) were only 20%. In cases of *Aeromonas* diarrhea, the most frequent associated bacteria corresponded to *Campylobacter* and *Salmonella*, while in cases of wound infections predominate other bacteria like *Klebsiella, Enterococcus, Escherichia coli, Staphylococcus aureus*, and *Yersinia enterocolitica* (Lamy et al., 2009; Chao et al., 2013; Zhou et al., 2019; Fernández-Bravo and Figueras, 2020; Yuwono et al., 2021; Robert et al., 2023). However, the interactions between the isolated strains of the different genera with *Aeromonas* have not yet been investigated. Previous studies demonstrated that the synergism between bacteria from different genera such as *Pseudomonas aeruginosa, S. aureus* or *Salmonella* increases the pathogenicity and the complexity of the disease (Nguyen and Oglesby-Sherrouse, 2016; Xu et al., 2019; Wilson et al., 2022). In this sense, polymicrobial infections tend to be associated with treatment failure and worse patient prognoses (Adamowicz et al., 2018; Little et al., 2021). Vega et al. (2013) demonstrated that the mixed culture of *E. coli* and *Salmonella Typhimurium* increases the tolerance to ciprofloxacin and in a study performed by Adamowicz et al. (2018) these mixed culture increased the tolerance to tetracycline.

Although *Y. enterocolitica* is a worldwide distributed agent of gastrointestinal infections, primarily affecting young individuals and commonly causing acute diarrhea (Hering et al., 2016; Nesbakken, 2021) like *Aeromonas* (Fernández-Bravo and Figueras, 2020). However, co-infections between *Aeromonas* spp. and *Yersinia* spp. in fecal samples are rarely reported (Latif, 2015; Hering et al., 2016; Nesbakken, 2021; Grave et al., 2022). In a study conducted in Australia, *Aeromonas* was the only enteropathogen in 1755 (82.3%) of the patients and out of the 377 patients that showed a mixed infection with another enterobacteria only 6 patients (1.4%) showed a coinfection with *Yersinia* (Yuwono et al., 2021). In another study performed, in Latvia, by Grave et al. (2022) about the prevalence of *Aeromonas* in a hospitalized pediatric population with gastroenteritis, 3 patients (6%) were affected by a co-infection of *Aeromonas* and *Y. enterocolitica*. In addition, from the unpublished data of the Ph.D. thesis of Latif (2015) only in one of the 409 patients studied a co-infection of *Aeromonas caviae* and *Y. enterocolitica* was observed. Recently, a new case of a patient that presented a gastrointestinal infection from which the same species, i.e., *A. caviae* and *Y. enterocolitica* were identified from the same feces culture was found. Considering the rarity of these cases and the lack of studies investigating the potential interaction between the bacteria of these genera, the present study aimed to evaluate the cell damage and immune response of macrophages after performing the single and dual infection with those strains. Furthermore, the antimicrobial susceptibility pattern of the single and mixed cultures was determined as well as the *in vitro* growth dynamics of the mixed strains.

## Materials and methods

### Isolation and identification

A stool sample from a patient with diarrhea was screened for bacterial pathogens, i.e., *Salmonella* spp., *Shigella* spp., *Campylobacter jejuni*, *Yersinia* spp., and diarrheagenic *E. coli* using specific culture media such as Cefsulodin-Irgasan-Novobiocin (CIN) agar, *Salmonella-Shigella* (SS), Campylosel agar, and MacConkey agar with sorbitol (SMAC) (bioMérieux®, France). Eight colonies were isolated from the CIN plates for preliminary identification. Subsequently, the Enterobacterial Repetitive Intergenic Consensus PCR (ERIC-PCR) was performed to identify specific fingerprint patterns among the isolates with the conditions previously described by Versalovic et al. (1991), using the primers referred to in Table 1. After the genotyping, two different isolates were primarily identified by Matrix-Assisted Laser Desorption/Ionization Time of Flight Mass Spectrometry (MALDI-TOF MS) with V3.1 of the Biotyper database (Bruker®). The cut-offs recommended were as follows: highly probable species level (scores ≥2.300), probable species level (scores 2.000–2.299), probable genus level (scores 1.700–1.999), and no reliable identification (scores ≤1.699). Both isolates were preserved in Tryptone Soya Broth (TSB) (Becton Dickinson GmbH, Germany) plus glycerol (20%) at −80°C. After growing the bacteria in Tryptone Soya Agar (TSA, Becton Dickinson GmbH, Germany) at 37°C for 24 h, the genomic DNA was extracted using the InstaGene™ DNA Purification Matrix (Bio-Rad Hercules CA, USA) following the manufacturer’s instructions (Fernández-Bravo et al., 2020). Their identity was determined after sequencing the *rpoD* and *16S rRNA* genes using primers and conditions previously described (Soler et al., 2004; Savin et al., 2014). A BLASTn analysis was conducted to find similarities between sequences. Subsequently, the sequences were aligned with 36 type strains of *Aeromonas* species and 12 strains of *Yersinia* species using the ClustalW algorithm, and the phylogenetic analysis was performed with the Neighbor-Joining (NJ) and maximum-likelihood algorithms (ML) in MEGA v6.0 (Fernández-Bravo and Figueras, 2020).

**Table 1:** Primers used in the study for ERIC-PCR and for gene expression analysis (Versalovic et al., 1991^*^; Murciano et al., 2015^#^; Zhao et al., 2017^+^).

Gene	Sequence (5′-3′)
ERIC-PCR^*^	ERIC 1R ATGTAAGCTCCTGGGATTCAC ERIC 2 AAGTAAGTGACTGGGGTGAGCG
*GAPDH^#^*	Forward CATGAGAAGTATGACAACAGCCT Reverse AGTCCTTCCACGATACCAAAGT
*TNF-*α^#^	Forward GAGGCCAAGCCCTGGTATG Reverse CGGGCCGATTGATCTCAGC
*CCL2^#^*	Forward CCCCAGTCACCTGCTGTTAT Reverse TGGAATCCTGAACCCACTTC
*CCL20^#^*	Forward GCAAGCAACTTTGACTGCT Reverse ATTTGCGCACACAGACAACT
*TP53^#^*	Forward CAGCACATGACGGAGGTTGT Reverse TCATCCAAATACTCCACACGC
*NLRP3^+^*	Forward CGTGAGTCCCATTAAGATGGAGT Reverse CCCGACAGTGGATATAGAACAGA
*IL-1B^+^*	Forward TTCGACACATGGGATAACGAGG Reverse TTTTTGCTGTGAGTCCCGGAG
*IL-6^#^*	Forward AACCTGAACCTTCCAAAGATGG Reverse TCTGGCTTGTTCCTCACTACT
*IL-8^#^*	Forward ACTGAGAGTGATTGAGAGTGGAC Reverse AACCCTCTGCACCCAGTTTTC

### Cell lines and culture conditions

The mouse macrophage BALB/c cell line J774A.1 (Tsuchiya et al., 1980) was selected for the infection experiments due to its significance as a model for studying the innate immune response against bacteria. This cell line was maintained as a cell adhesion in Dulbecco’s Modified Eagle’s Medium (DMEM, PAA Laboratories GmbH, Germany) supplemented with 10% FBS (fetal bovine serum, PAA Laboratories) plus 1% P/S solution (penicillin–streptomycin, PAA Laboratories) at 37°C and 5% CO_2_. Prior to infection experiments, cells were seeded in tissue culture plates containing DMEM without FBS and P/S (serum and antibiotic-free conditions) at a concentration of 0.5 × 10^6^ cells/mL to obtain 1 × 10^6^ cells/mL after 24 h (Murciano et al., 2017).

### Infection

Before the infection, bacteria were grown at 37°C in serum and antibiotic-free DMEM under shaking conditions (100 rpm) for 24 h. Cell line J774A.1 was infected with the individual strains of *A. caviae* and *Y. enterocolitica* using overnight cultures at multiplicity of infection (MOI) of 10, i.e., the ratio between the number of bacteria and the number of targeted cells (Fernández-Bravo and Figueras, 2022). For the polymicrobial studies, the bacterial strains were mixed in a ratio 1:1 and the cells were infected at MOI 10. The infected cell lines were then incubated at 37°C and 5% CO_2_ and sampled at specific times depending on the experiment.

### Cell damage assay (lactate dehydrogenase assay)

After infection with the single and the mixed strains at MOI 10, supernatants were collected at 4 h and 6 h post-infection (Fernández-Bravo and Figueras, 2022). Cell damage was determined by measuring the lactate dehydrogenase (LDH) enzyme released into the supernatants, indicating plasma membrane damage. The Cytox 96 Non-Radioactive Cytotoxicity Assay (Promega, Madison, WI, USA) was performed according to the manufacturer’s instructions and read with the SPECTRA spectrophotometer (Biotek, Spain). A standard curve was generated using bovine recombinant LDH (Sigma-Aldrich, St Louis, MO, USA). LDH levels in the samples were determined by extrapolation from the curve (Murciano et al., 2015; Fernández-Bravo and Figueras, 2022).

### MTT assay

The MTT Cell Proliferation Assay 30-1010K™ (ATCC) was used as a measure of macrophage viability after infection with the mixed ant the single cultures. Macrophages were seeded in 96-well microtiter plates. Then, macrophages were infected at MOI 10 and subsequently incubated with gentamicin or streptomycin (50 μg/mL) for 45 min (Fernández-Bravo and Figueras, 2022). Later, the medium was replaced with fresh DMEM for 6 h at 37°C and 5% CO_2_. After incubation, the MTT reagent was added to the wells (10 μL/well), and microplates were incubated at 37°C and 5% CO_2_ for 2 h. At that point, 100 μL of the detergent reagent was added following incubation in darkness at room temperature for 2 h (Andersson et al., 2016). Finally, absorbance was measured at 570 nm in a Spectramax M5e microplate reader (Molecular Devices, Sunnyvale, CA, USA). Viability percentages were calculated with respect to non-infected macrophages incubated with DMEM, for which a value of 100% of viability was established.

### Intracellular survival

Monocytes infected at MOI 10 were incubated at 37°C with 5% CO_2_ for 1 h. Subsequently, gentamicin or streptomycin treatment (50 μg/mL) for 1 h was administered to eliminate extracellular bacteria (time 0), after which the bacterial count within the monocytes was assessed (Fernández-Bravo and Figueras, 2022). The infected monocytes were then incubated further with fresh DMEM and a maintenance dose of gentamicin or streptomycin (2 μg/mL) for an additional 4 h. Following this, the bacterial count within J7741.A cells was determined. Percent survival was calculated based on the bacterial count after 4 h of incubation and after gentamicin or streptomycin treatment (50 μg/mL) (Fernández-Bravo and Figueras, 2022).

### Analysis of the expression of the genes related to the immune system

Six different genes implicated in the immune response against pathogens were selected to quantify their transcription levels in macrophages (J774A.1) in response to the infections produced with the single and mixed strains in relation to the non-infected cells. The primers used to evaluate the expression of the selected genes were those from Murciano et al. (2017) and Zhao et al. (2017) and are listed in Table 1. The selected genes encoded for: cytokines and chemokines (*TNF- α, IL-6, IL-8, CCL2* and *CCL20*), apoptosis (*TP53*) and pyroptosis (*NLRP3* and *IL-1β*). After 4 h of infection at MOI 10, J774A.1 cells were washed twice with PBS, and the RNA was isolated from the samples by using the GenElute^™^ Mammalian Total RNA Miniprep Kit (Sigma-Aldrich). The cDNA was transcribed from total RNA by using the iScript cDNA Synthesis Kit (Bio-Rad Laboratories, Inc. Hercules, CA, USA). A Real-time PCR was performed with cDNA for quantification by using the Power SYBR® green PCR Master Mix (Applied Biosystems®, Life Technologies, Glasgow, UK) on a StepOnePlus™ Real-Time PCR System (Applied Biosystems®). The thermal cycling conditions were: 94°C for 5 min, followed by 40 cycles of 30s at 94°C, 30s at 61°C, 30s at 72°C, and finally 20s at 80°C. Threshold cycle (CT) values were obtained to establish the relative RNA levels of the tested genes, using the glyceraldehyde-3-phosphate dehydrogenase (*GAPDH*) gene as a housekeeping gene of reference. The relative gene expression was determined by using the delta–delta Ct (2–∆∆Ct) method that relays on the signal from the real-time polymerase chain reaction, as done in previous studies (Murciano et al., 2015; Fernández-Bravo and Figueras, 2022).

### Dynamic growth: *in vitro* contact killing assay

Overnight cultures of both strains were mixed in a ratio 1:1, spotted on nonselective LB agar plates, and incubated for 4 h at 37°C. The number of each strain before and after incubation for 4 h in mixtures was determined by serial dilution and plating on bacterial selective medium ampicillin dextrin agar (ADA) and CIN for *Aeromonas* and *Yersinia*, respectively, (Fernández-Bravo et al., 2019). Percent survival was obtained based on the number of bacteria after 4 h of incubation in relation to the number before incubation (Murciano et al., 2017).

### Antimicrobial susceptibility testing

*In vitro* antimicrobial susceptibility testing of both single strains and mixed cultures was determined by BD BBL™ Sensi-Disc™ (Oxoid, London, UK). The commercial antibiotics used for *Aeromonas* and *Yersinia* infections and other included as controls were ciprofloxacin (30 μg), trimethoprim (25 μg), piperacillin-tazobactam (100/10 μg), chloramphenicol (10 μg), penicillin (10 μg), cefepime (30 μg), amikacin (30 μg), amoxicillin-clavulanic (20/10 μg), imipenem (10 μg), ampicillin (10 μg), gentamicin (10 μg), aztreonam (30 μg), cefotaxime (30 μg), cephalotin (30 μg), ceftazidime (30 μg), tobramycin (10 μg), and ertapenem (10 μg). First, a fresh culture with a turbidity of 0.5 McFarland was swabbed onto the surface of Mueller-Hinton agar (MHA) (HiMedia, Mumbai, India). For the mixed culture of *Aeromonas* and *Yersinia* the concentration was calculated 1:1 with a final turbidity of 0.5 McFarland. The antibiotic disks were placed on the MHA surface using sterile forceps, and the agar plates were incubated at 37°C for 24 h. The inhibitory zones were interpreted according to the measurements provided by both the Clinical and Laboratory Standards Institute (CLSI, 2020) and the European Committee on Antimicrobial Susceptibility Testing Guidelines (EUCAST, 2021). Finally, as a control, the amount of each strain in the co-culture with the antibiotic gentamicin, after the antimicrobial test, was determined by collecting all the bacteria, performing a serial dilution and plating on selective bacterial medium ampicillin dextrin agar (ADA) and CIN for *Aeromonas* and *Yersinia*, respectively, (Fernández-Bravo et al., 2019).

### Statistical analysis

All the experiments were performed in triplicate and the statistical significance was determined by using the Student’s two-tailed *t*-test or two-way ANOVA at *p* < 0.05 using the GraphPad Prism 6.0 (GraphPad Software, CA, USA). Analysis was chosen based on the type of experiment.

## Results

### Isolation and identification

The preliminary identification of the strain 1185C with the MALDI-TOF Biotyper, considering exclusively the first match in the identification and applying manufacturer recommended cut-off, resulted in a score lower than 2.0 for *A. hydrophila* (1.893) and higher for *A. caviae* (2.051). The strain 1186C showed a higher score than 2.0 for *Y. enterocolitica* (2.121). A BLASTn analysis using the obtained *rpoD* and *16S rRNA* sequences revealed 99% similarity with a strain of *A. caviae* (accession number PP404053) and *Y. enterocolitica* (accession number PP280108), respectively. Furthermore, the phylogenetic trees constructed with the *rpoD* and *16S rRNA* gene of these strains and the sequences of the type strains of *Aeromonas* spp. and *Yersinia* spp. revealed that the sequences of the isolated strains clustered with the sequences of the type strains of *A. caviae* and *Y. enterocolitica* (Supplementary Figures 1, 2).

### Cell damage and intracellular survival on macrophages after the single and mixed infections

**Figure 1:**
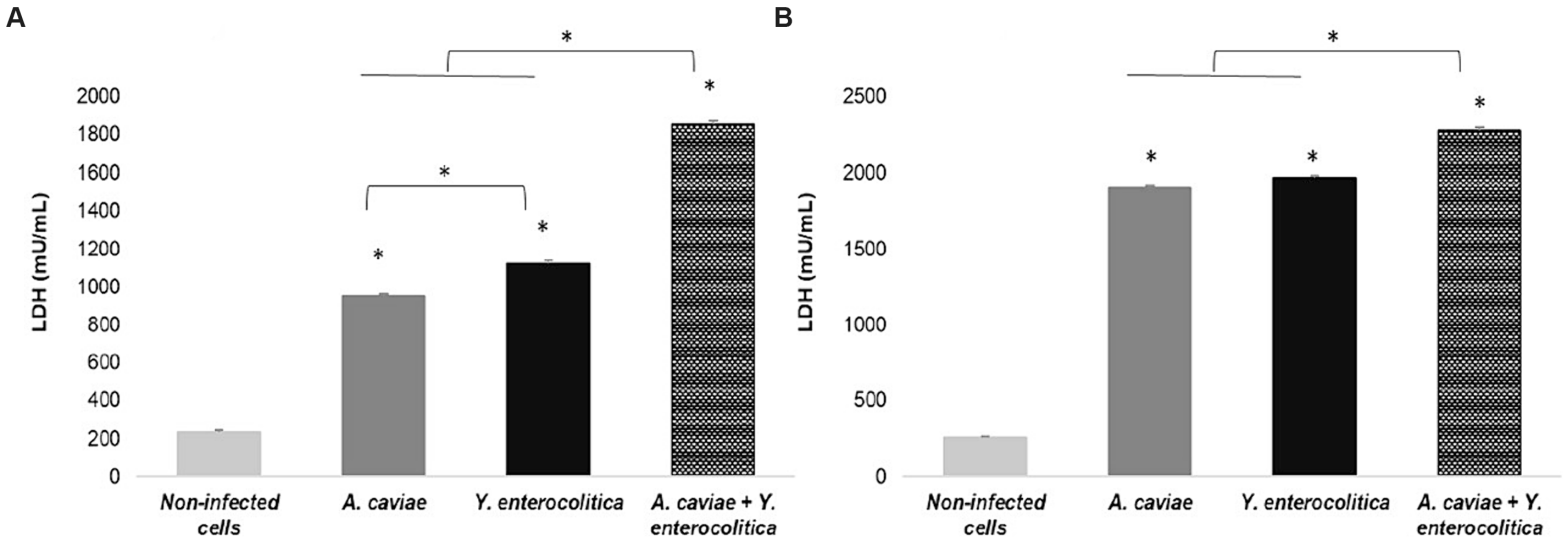
Cell damage in J774A.1 macrophages induced at 4 h **(A)** and 6 h **(B)** by the single and mixed infection at MOI 10 in relation to the non-infected cells, measured by the release of lactate dehydrogenase (LDH) enzyme. Asterisks indicate statistically significant difference *p < 0.05.

The capacity of the single and mixed infections to induce cell damage in macrophages (J774A.1) was evaluated through the release of LDH to the cell culture supernatant is shown in Figure 1. All infections, regardless of mixed or single at MOI 10, resulted in a significant degree of cell damage at 4 h (*p* < 0.05) when compared to the non-infected cells. Furthermore, it was found that the degree of cell damage increased at 6 h (*p* < 0.05) (Figure 1). The strain of *Y. enterocolitica* (1186C) was able to induce a higher degree (*p* < 0.05) of cell damage than the strain of *A. caviae* (1185C) at 4 h (Figure 1A). However, no significant difference in cell damage between *Y. enterocolitica* and *A. caviae* was observed at 6 h (Figure 1B). In addition, co-infection with both strains 1185C and 1186C caused significantly higher cell damage than the single infection, independently of the exposure time (*p* < 0.05) (Figure 1).

**Figure 2:**
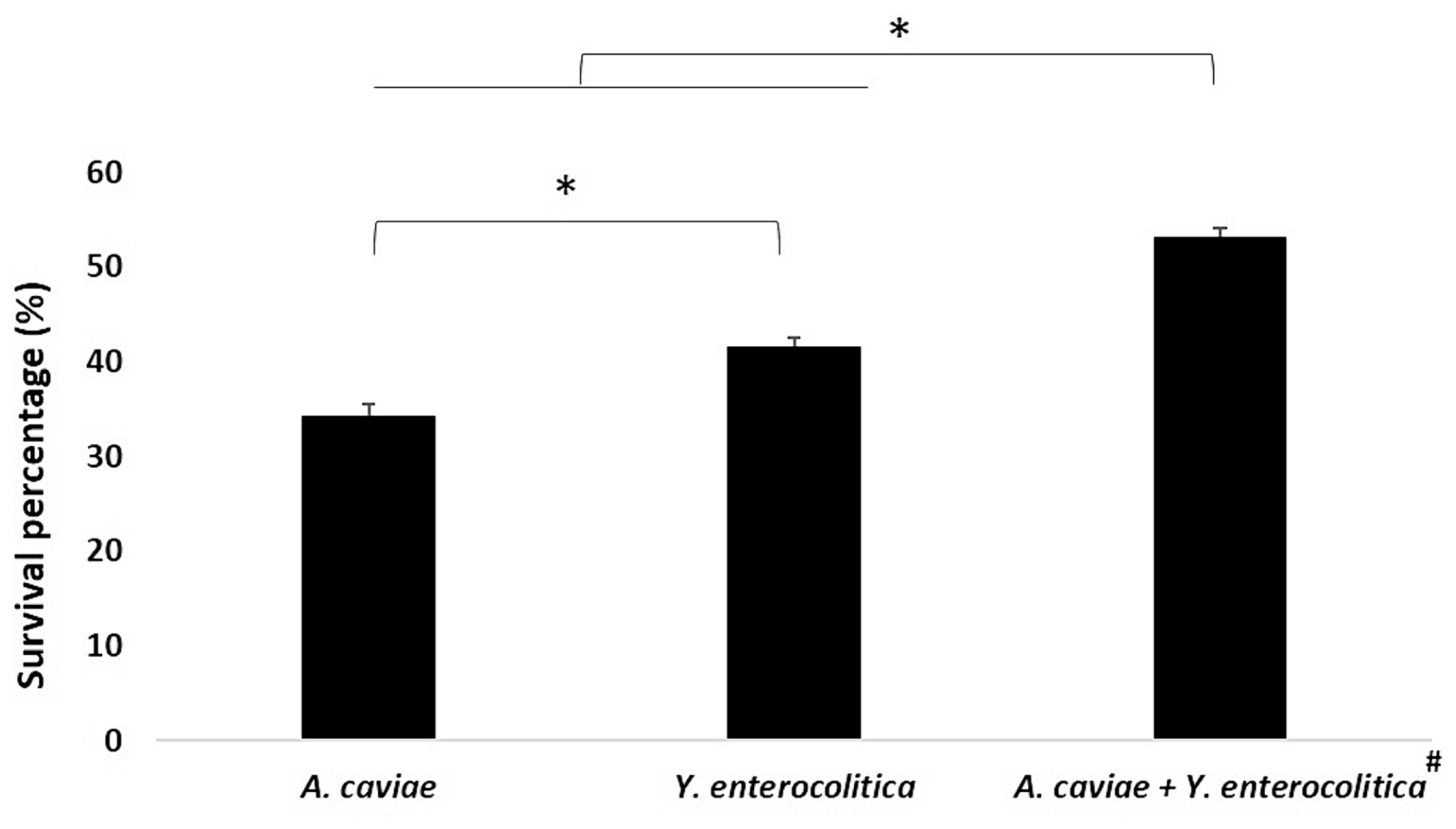
Intracellular survival at 4 h in J774A.1 macrophages after the single and mixed infections at MOI 10. Asterisks indicate statistically significant difference *p < 0.05. The viability of *Yersinia* (32.11%) was higher than that *Aeromonas* (21.10%)^#^.

As shown in Figure 2, the mixed infection at 4 h with *Y. enterocolitica* 1186C and *A. caviae* 1185C at MOI 10 showed a significant higher intracellular survival (*p* < 0.05) with a 53.21% of survival percentage than the single infections. The viability in the mixed culture of *Yersinia* was 32.11% being higher than that of *Aeromonas* (21.10%). The percentage of survival with the single infections was higher (*p* < 0.05) after *Y. enterocolitica* (41.54%) infection in comparison with *A. caviae* (34.32%).

### Viability of J7741.A macrophages after single and mixed infection

**Figure 3:**
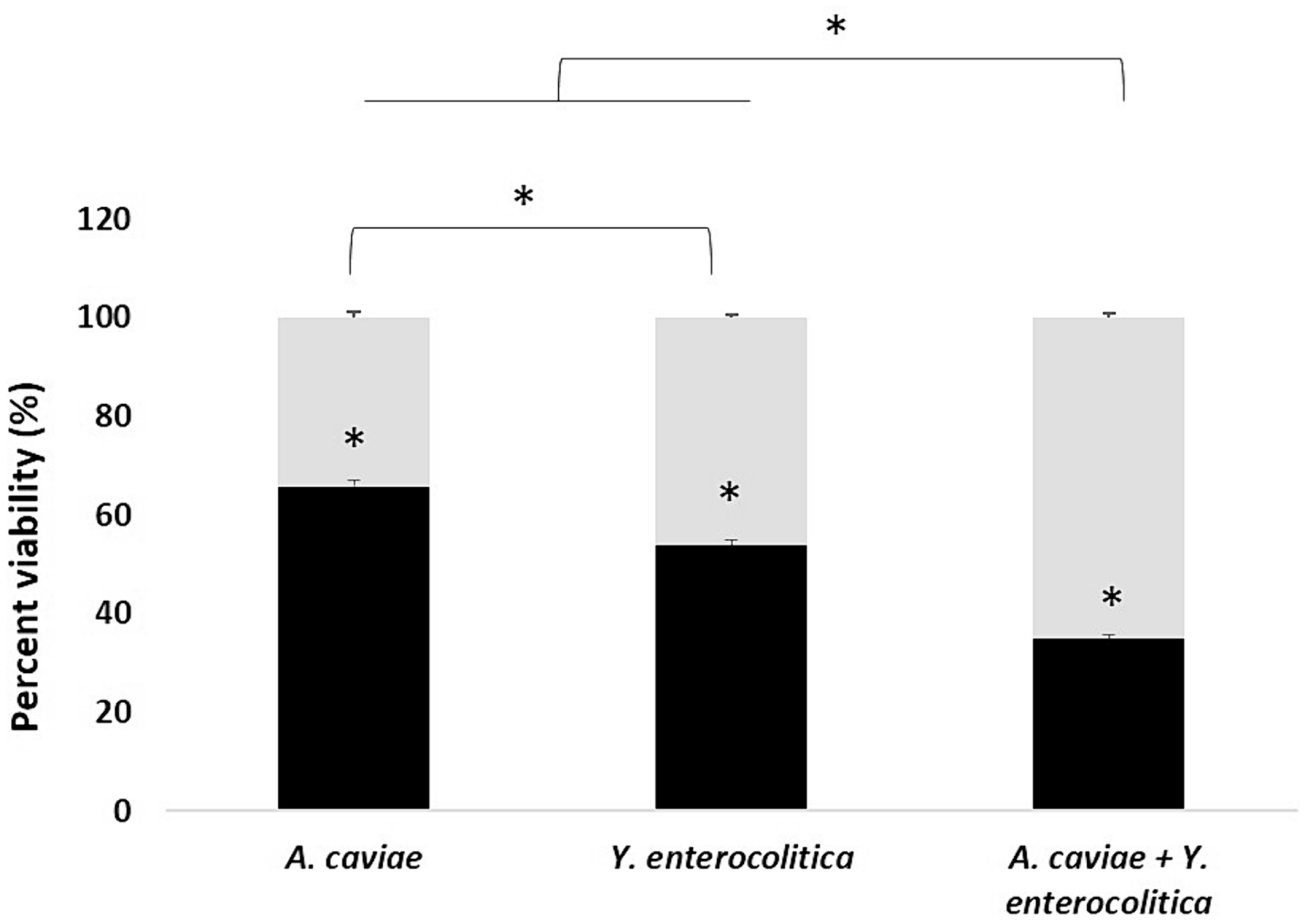
Viability percentage of J774A.1 macrophages at 5 h after infection with the single and mixed infection at MOI 10. Asterisks indicate statistically significant difference *p* < 0.05*.

The results on viability of infected macrophages incubated with different single and mixed cultures, measured as the reduction of MTT, are shown in Figure 3. Infected macrophages with *Y. enterocolitica* (1186C), showed a significant reduction in their viability (*p* < 0.05), 100 to 53.87% in relation to *A. caviae* (1185C), 100 to 65.78%. The highest level of viability was observed for macrophages incubated with the mixed culture (34.89%).

### Expression of the immune-related genes caused by the single and mixed infection

#### Genes for cytokines and chemokines

**Figure 4:**
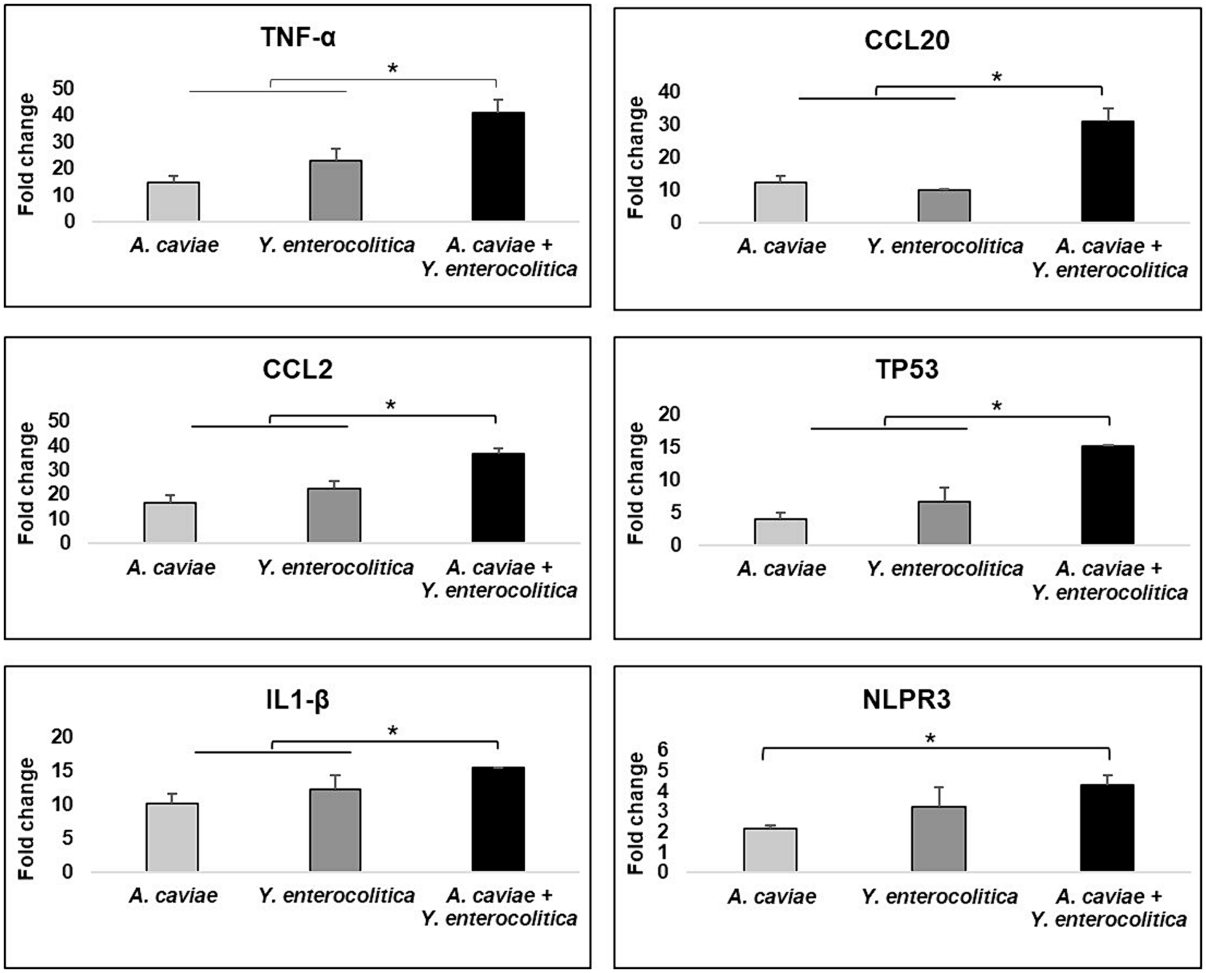
Gene expression profile of J774A.1 macrophages in relation to the non-infected cells induced by the single and mixed infections at MOI 20 d1termined by RT-qPCR. Transcript levels of the genes were normalized to the expression of the *GAPDH* gene. Expression fold change respect to the non-infected cells was calculated using the comparative ∆∆Ct method. Asterisks indicate statistically significant difference *p < 0.05.

All macrophage infections induced the expression of the genes encoding cytokine *TNF-α* and chemokines (*CCL2, CCL20*), with a significant increase (*p* < 0.05) in the transcript expression levels in relation to the non-infected cells (Figure 4). In addition, the *TNF-α* and *CCL2* gene expression levels were significantly higher after infection with the *Y. enterocolitica* strain 1186C in comparison with the *A. caviae* strain 1185C. However, the expression of the *CCL20* gene was significantly higher after infection with the latter strain (1185C) than with the *Y. enterecolitica* (1186C) infection (*p* < 0.05) (Figure 4). The transcriptional level after the macrophage infections with the mix of both strains was higher for the three genes *TNF-*α, *CCL2* and *CCL20* in comparison to the single infections (*p* < 0.05). However, the expression level of the proinflammatory cytokine genes IL-6 and IL-8 was close to the limit of detection for all single and mixed infections and no significant differences were found compared to non-infected cells (Supplementary Figure 3).

#### Gene involved in apoptosis

The transcriptional level of *TP53* apoptosis gene increased after infection with all single and mixed cultures (*p* < 0.05) (Figure 4). The expression of the *TP53* gene showed no significant differences between the single infections with *Y. enterocolitica* 1186C and *A. caviae* 1185C (Figure 4). However, the expression levels of *TP53* were significantly higher after the infection was produced with the mix of both strains in comparison with the single infections (*p* < 0.05) (Figure 4).

#### Genes related to the inflammasome and pyroptosis

The *NLRP3* and *IL1-β* genes which are associated with pyroptosis, i.e., cell death mediated by the formation of the inflammasome, were overexpressed in J774A.1 cells in response to all infections when compared with the non-infected cells (*p* < 0.05) (Figure 4). The expression levels of both genes were significantly higher after the single infection with *Y. enterocolitica* strain 1186C in comparison with *A. caviae* strain 1185C. The upregulation of *IL-1β* showed an increase (*p* < 0.05) when J774A.1 cells were infected with a mixed culture of both strains. No significant differences in the level of the *NLRP3* expression were observed when comparing the mixed infection with the *Y. enterocolitica* single infection. However, significant differences in the expression of the *NLRP3* were detected (*p* < 0.05) for the mixed infection in comparison with the *A. caviae* single infection (Figure 4).

### *In vitro* dynamics of co-cultures

**Figure 5:**
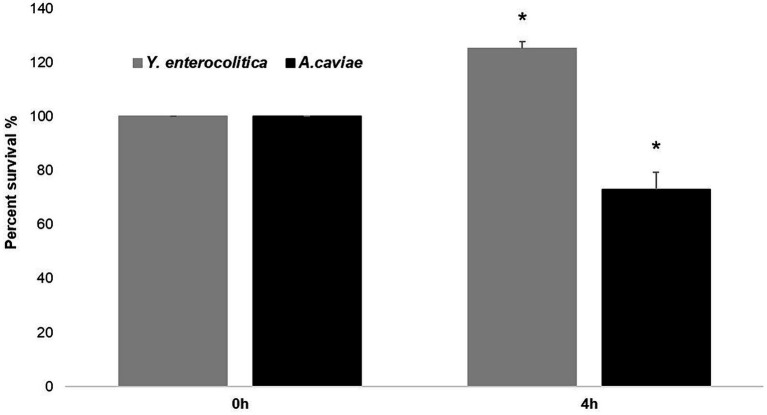
Killing effect of *Y. enterocolitica* to *A. caviae* after mixed culture during 4 h in a ratio of 1: 1. Asterisks indicate statistically significant difference *p < 0.05.

When the *A. caviae* strain 1185C was mixed 1:1 with the *Y. enterocolitica* strain 1186C during 4 h and plated on ADA and CIN agar, the colony count for *A. caviae* strain 1185C decreased from 100 to 73% (Figure 5). However, the number of colonies for the strain *Y. enterocolitica* 1186C increased from 100 to 127% in mixed culture after 4 h (Figure 5).

### Antimicrobial susceptibility patterns

The antimicrobial susceptibility results showed that individually, the *A. caviae* 1185C and the *Y. enterocolitica* 1186C strains were resistant to penicillin, amoxicillin/clavulanic, ampicillin and cephalothin and with an intermediate resistance to trimethoprim. For the other antibiotics, these strains were sensible (Table 2). However, the co-culture of both strains showed a different antimicrobial pattern in comparison with the single strains for ciprofloxacin, trimethoprim, chloramphenicol, gentamicin and ertapenem, increasing the resistance to these antibiotics. The results of co-cultures after gentamicin treatment showed that the inhibition halos overlapped between *Aeromonas* and *Yersinia* and the amount of bacteria in relation to the total colonies was 37.15% and 62.85%, respectively.

**Table 2:** Antimicrobial susceptibility pattern (mm).

Antimicrobial agent	*A. caviae*	*Y. enterocolitica*	*A. caviae* + *Y. enterocolitica*
Ciprofloxacin	S (22.12)	S (23.21)	I (16.05)
Trimethoprim	I (16.23)	I (17.13)	R (8.52)
Piperacillin/tazobactam	S (18.32)	S (17.17)	S (21.35)
Chloramphenicol	S (19.23)	S (21.45)	R (14.12)
Penicillin	R (12.34)	R (13.53)	R (12.78)
Cefepime	S (29.32)	S (27.34)	S (28.54)
Amikacin	S (21.65)	S (19.41)	S (22.14)
Amoxicillin/clavulanic	R (12.56)	R (11.45)	R (4.56)
Imipenem	S (28.64)	S (29.41)	S (28.54)
Ampicillin	R (11.45)	R (8.45)	R (9.45)
Gentamicin	S (17.56)	S (19.43)	R (7.34)
Aztreonam	S (23.45)	S (24.23)	S (23)
Cefotaxime	S (28.65)	S (27)	S (29.41)
Cephalotin	R (2.36)	R (4.56)	R (3.14)
Ceftazidime	S (29.56)	S (28.14)	S (27.41)
Tobramycin	S (17.25)	S (19.45)	S (21.14)
Ertapenem	S (30.41)	S (28.51)	I (26)

S, sensible; I, intermediate; R, resistant.

## Discussion

The infections caused by *Aeromonas* can involve more than one strain or strains belonging to *Aeromonas* or other genera (Lamy et al., 2009; Chao et al., 2013; Barraud et al., 2020; Lin et al., 2020; Vithiya and Raja, 2022). However, to the best of our knowledge, very few studies have investigated the potential interactions of the isolated bacteria. Mosser et al. (2015), Fernández-Bravo et al. (2019), and Robert et al. (2023) showed that the presence of two *Aeromonas* strains can sometimes increase the virulence in the host. Therefore, the interest of this study resides in evaluating whether a mixed infection between *A. caviae* and *Y. enterocolitica* isolated from the same patient can increase virulence and what are the differences in terms of antimicrobial susceptibility.

Our results showed that *Y. enterocolitica* induces higher cell damage on macrophages than *A. caviae* after 4 h of infection. However, the cell damage was higher after co-infecting the macrophages with a mixed culture of both bacteria. Therefore, the dual infection with *A. caviae* and *Y. enterocolitica* increases the pathogenicity. Consistent results were obtained in the MTT assay being the highest survival percentage after single infection in relation to the mixed infection with both bacteria. Furthermore, intracellular survival was higher after infection with *Y. enterocolitica* compared to *A. caviae*, and the survival rate was higher after mixed infection. These results agree with those obtained by Mosser et al. (2015) that demonstrated using the *Caenorhabditis elegans* animal model an increase of the virulence after infection with two *Aeromonas* strains in relation to a single strain, indicating a synergism between both strains. In a recent study performed by Barraud et al. (2020) in which they investigate the behavior of two strains of *Aeromonas veronii*, recovered from an infected flap after leech therapy, all the analyses showed that the virulence of both recovered strains did not differ when tested individually but virulence was enhanced in the mixed infection. The authors showed that their results suggest that the outcome of the infection could have been worsened due to the synergies produced by the two isolates. However, Robert et al. (2023) using *Drosophila melanogaster* as a model, detected various effects depending on the strains used for co-cultures, with virulence sometimes more significant and others less or the same. Xu et al. (2019) demonstrated that the presence of polymicrobial infections between *Pseudomonas aeruginosa* and *Staphylococcus aureus* induces biofilm formation, increasing the level of pathogenicity. The recent literature review has shown an increase in virulence factor production by *P. aeruginosa* in a polymicrobial environment with S. aureus (Little et al., 2021). Some *Yersinia* and *Aeromonas* species have type three secretion systems (T3SS), and both are capable of inflicting gastroenteritis via the fecal-oral route of infection (Frey and Origgi, 2016; Romero et al., 2016; Mandal et al., 2017). This T3SS acts as a vehicle that can inject or translocate different toxins in the host immune cells and increase virulence (Rosenzweig and Chopra, 2013; Rangel et al., 2019). Therefore, considering the relevance of this finding, further studies must be carried out to elucidate the responsible mechanism of such behavior.

In relation to the expression of immune-related genes, higher transcriptional levels were observed after mixed infection in comparison with the single infections, except for the NLRP3 pyroptosis gene that recognizes pathogen-associated molecular patterns (PAMPs) to induce the activation of the immune response (Kumar et al., 2011). In a previous study using the monocytic human cell line THP-1 as *in vitro* infection model, Fernández-Bravo and Figueras (2022) investigated the innate human immune response against six commonly found clinical *Aeromonas* species, evaluating cell damage, intracellular survival and the expression of 11 immune-related genes and results demonstrated that *A. caviae* induce a cytokine storm that resulted in an increase in virulence. In the present study, results suggest that after the mixed infection, the virulence would increase due to the overexpression of the immune-related genes. In addition, this previous study suggested that different pathways of cell death were activated depending on the *Aeromonas* species. For example, in *A. caviae* both routes (apoptosis and pyroptosis) were induced, as it was shown to occur in the present study by the overexpression of TP53 and NLRP3 genes. These findings on the immune response are very interesting since they reinforce the hypothesis that the innate immune response is species-specific for *Aeromonas*, since the results of this study with *A. caviae* are similar to those obtained in the previous study by Fernández-Bravo and Figueras (2022).

The antimicrobial patterns of the bacteria varied when they were analyzed in pure cultures or in mixed cultures. Both single bacteria were susceptible to ciprofloxacin and ertapenem, however, thesusceptibility was intermediate in the mixed culture. In addition, the single cultures were susceptible to trimethoprim, chloramphenicol, and gentamicin, while the mixed cultures were resistant to these antibiotics. This is relevant because some of these antibiotics, i.e., ertapenem, trimethoprim, or ciprofloxacin are used empirically for the treatment of *Aeromonas* and *Yersinia* and this may hamper the effectivity of the treatment (González-Torralba et al., 2018; Dhanapala et al., 2021). These results are in agreement with previous studies of polymicrobial infections between other bacterial genera. In fact, Vega et al. (2013) demonstrated that the mixed culture of *E. coli* and *S. typhimurium* increases the tolerance to ciprofloxacin as occurred in our study. Our hypothesis about this increase in tolerance is that in polymicrobial infections Quorum-Sensing would have a role in inducing the expression of different genes, as well as increasing the production of enzymes and even the formation of a barrier that reduces the permeability of drugs.

The growth dynamics in our study showed that when the strains were mixed for the contact-killing assay in LB, colony counts of *Y. enterocolitica* increased (and the *A. caviae* decreased at 4 h). This result could be associated with the antagonism of both bacteria as occurred in a previous study with two strains of *A. hydrophila* in which one eliminated the other (Fernández-Bravo et al. 2019). In the latter study, the type six secretion system (T6SS) of one strain produced the TseC effector toxin that directly killed the other *in vitro* and *in vivo*. Our results in the present study could be associated with the presence and the overexpression of the T6SS in *Y. enterocolitica*. Ongoing studies (unpublished) have demonstrated the presence of genes that encode different proteins related to the T6SS by PCR in both strains. However, further studies to clarify the functionality of this T6SS by transcriptomic analysis are necessary to prove this suspicion.

To our knowledge, this is the first study that demonstrates the interactions between co-isolated bacteria of *Aeromonas* with other different genera associated with a diarrheal process and their potential impacts on the evolution of the disease and its treatment. This study shows the need for performing further studies to better understand the complexity of occurring mixed infections and pretends to alert clinicians of the importance to take into account the potential interactions among these bacteria in relation to the treatment efficacy and outcome of the disease.

## Conclusion

Mixed infection with *A. caviae* and *Y. enterocolitica* induces differences in the activation of the immune response in macrophages compared with single infections, as well as an increase in the cell damage that may enhance the infection disease process. Apparently, both synergistic and antagonistic effects may coexist as well as an increase in the antimicrobial resistance of the antimicrobials used to empirically treat infections produced by these bacteria. The latter may potentially hamper antimicrobial treatment. This study indicates for the first time the importance of performing further studies on *Aeromonas* mixed infections to clarify the specific mechanisms of those interactions and their impact on the evolution of infectious diseases.

## Data availability statement

The datasets presented in this study can be found in online repositories. The names of the repository/repositories and accession number(s) can be found in the article/Supplementary material.

## Author contributions

AF-B: Data curation, Formal analysis, Investigation, Methodology, Writing – original draft, Writing – review & editing. GR: Data curation, Methodology, Writing – review & editing. MF: Conceptualization, Funding acquisition, Project administration, Resources, Supervision, Writing – original draft, Writing – review & editing.
