# Supplementary material for: Interactions between Aeromonas caviae and Yersinia enterocolitica isolated from a case of diarrhea: evaluation of antimicrobial susceptibility and immune response of infected macrophages
Source: Front Microbiol. 2024 Apr 24;15:1328766. doi: 10.3389/fmicb.2024.1328766 (PMC11076857; doi:10.3389/fmicb.2024.1328766)
Supplement: Supplementary file 1 [file Data_Sheet_1.pdf]

SUPPLEMENTARY MATERIAL

Interactions between *Aeromonas caviae* and *Yersinia enterocolitica* isolated from a case of diarrhea and evaluation of their antimicrobial susceptibility and immune response of infected macrophages.

Ana Fernández-Bravo[a,b*], Gemma Recio[a,b,c], and Maria José Figueras [a,b*]

[a]Department of Basic Medical Sciences, Rovira i Virgili University, 43201Reus, Spain.

[b]Pere Virgili Health Research Institute (IISPV), Reus, Spain

[c]Laboratori Clínic ICS Camp de Tarragona-Terres de l'Ebre, Instituto Catalán de la Salud, Tarragona, Spain.

Corresponding author: mariajose.figueras@urv.cat; ana.fernandez@urv.cat

Keywords: *Aeromonas caviae, Yersinia enterocolitica*, mixed infections, macrophage, immune response

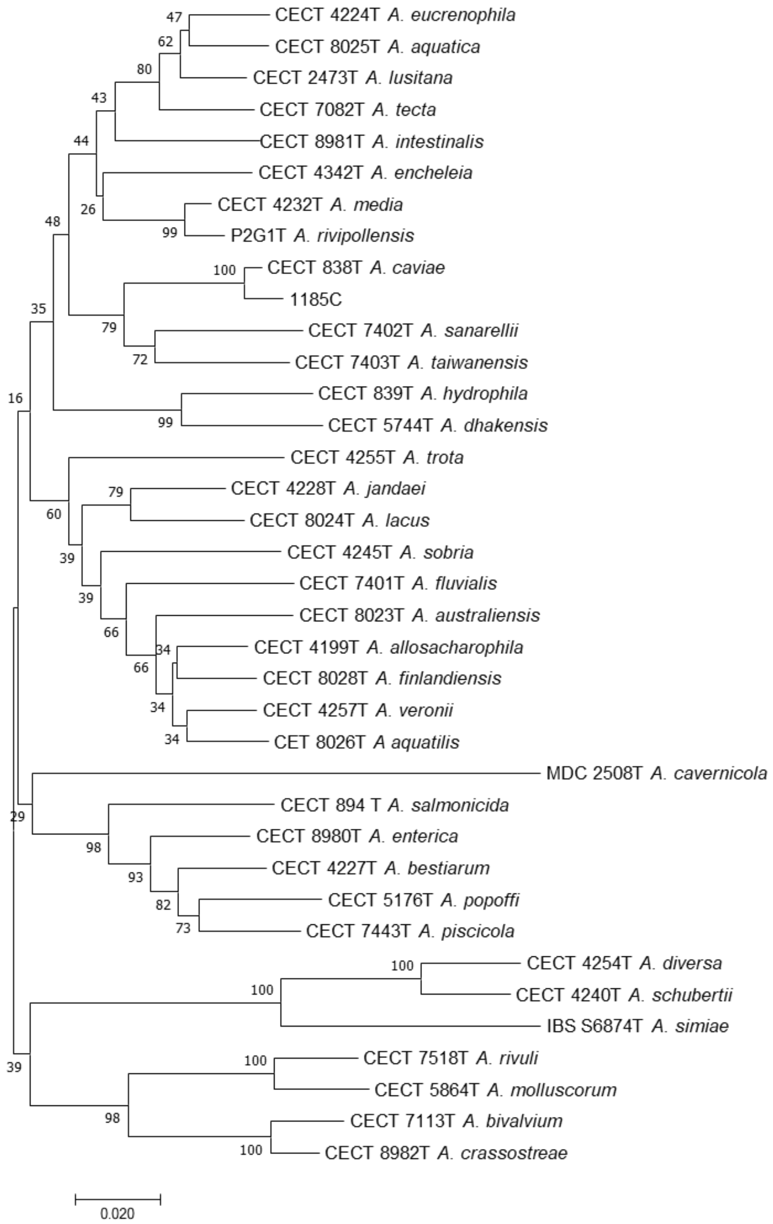

Supplementary Fig 1. Neighbor joining phylogenetic tree of the genus *Aeromonas* (459 bp) based on *rpoD* sequence alignments. Bootstrap support values are shown close to the branches.

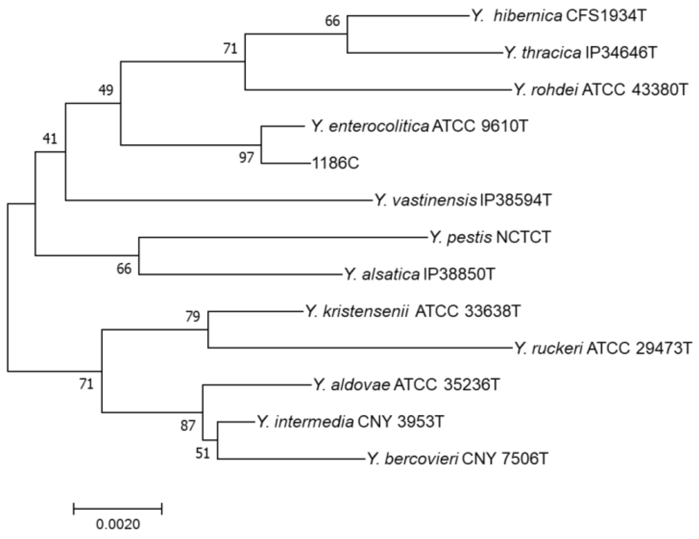

Supplementary Figure 2. Maximum-likelihood phylogenetic tree of the genus *Yersinia* (1443 bp bp) based on *16S rRNA* sequence alignments. Bootstrap support values are shown close to the branches. Bar, 0.01 amino acid substitutions per character.

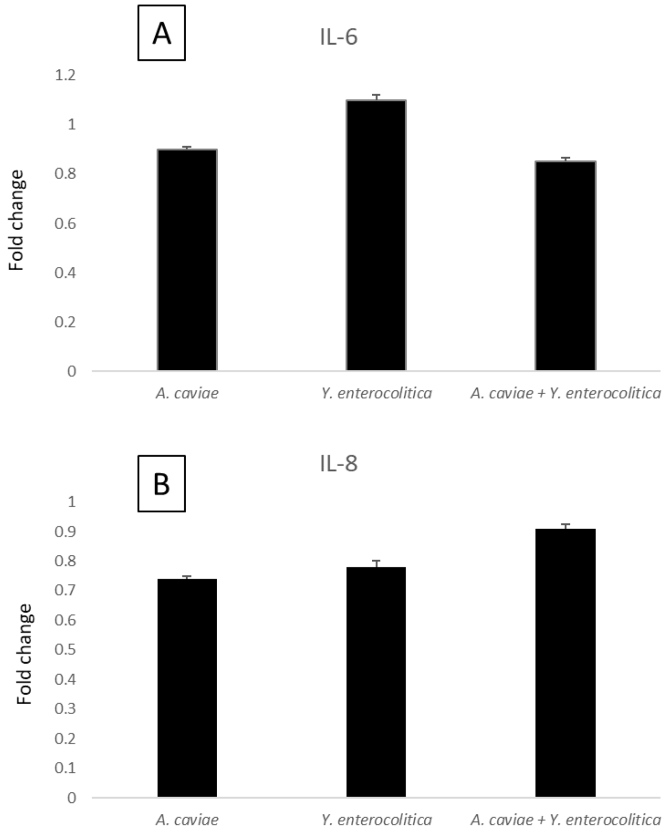

Supplementary Figure 3. *IL-6* (A) and *IL-8* (B) gene expression profile of J7741.A cells in relation to the non-infected cells induced by the different studied single and mixed infections. The expression levels were below the detection limit for all species.
